# Sentinel Trees as a Tool to Forecast Invasions of Alien Plant Pathogens

**DOI:** 10.1371/journal.pone.0120571

**Published:** 2015-03-31

**Authors:** AnnaMaria Vettraino, Alain Roques, Annie Yart, Jian-ting Fan, Jiang-hua Sun, Andrea Vannini

**Affiliations:** 1 DIBAF, University of Tuscia, Viterbo, Italy; 2 INRA-UR633, Zoologie Forestière, Centre de recherche d'Orléans, Orléans, France; 3 School of Forestry and Bio-technology, Zhejiang A & F University, Lin'an, China; 4 State key laboratory of Integrated Management of pest Insects and Rodents, Institute of Zoology, Chinese Academy of Sciences, Beijing, China; Virginia Tech, UNITED STATES

## Abstract

Recent disease outbreaks caused by alien invasive pathogens into European forests posed a serious threat to forest sustainability with relevant environmental and economic effects. Many of the alien tree pathogens recently introduced into Europe were not previously included on any quarantine lists, thus they were not subject to phytosanitary inspections. The identification and description of alien fungi potentially pathogenic to native European flora before their introduction in Europe, is a paramount need in order to limit the risk of invasion and the impact to forest ecosystems. To determine the potential invasive fungi, a sentinel trees plot was established in Fuyang, China, using healthy seedlings of European tree species including *Quercus petreae*, *Q*. *suber*, and *Q*. *ilex*. The fungal assemblage associated with symptomatic specimens was studied using the tag-encoded 454 pyrosequencing of the nuclear ribosomal internal transcribed spacer-1 (ITS 1). Taxa with probable Asiatic origin were identified and included plant pathogenic genera. These results indicate that sentinel plants may be a strategic tool to improve the prevention of bioinvasions.

## Introduction

In the past 200yrs, the number of fungal and fungal-like Invasive Alien Species (IAS) challenging plants in Europe has increased exponentially, especially in regards to ascomycetes [1]. Most of them (57%) have been introduced to Europe through living plants and have caused serious epidemics. The recent outbreak of ash dieback in Central Europe and the UK [2], the spread of pitch canker of pine in plantations in Spain [3], the sudden appearance of *Phytophthora* spp. outbreaks in the UK [4]-[5] challenging both plantations and natural forests are only recent examples of new threats to be added to ‘old’ invasions still threatening European forests.

The temporal trend of invasion in Europe reflects the history of trading living plants and associated commodities between Europe and other countries. After World War I, the commercial exchanges with North America increased as well as the number of invasive pathogens from USA [1]. The proliferation of pathogens from Asia reflects the trend in trading of living plants, especially between Europe and China [6].A common feature of invasive pathogens responsible for epidemics in forests is that most of them were first described (in Europe or elsewhere) after they had escaped from their geographic centre of origin and begun causing damage in a susceptible plant community. In other words, they were unknowns to science before invasion.

Most of the National Plant Protection Organizations (NPPOs) regulate the inspections of living plants and commodities based on lists of known organisms described as invasive elsewhere.

Furthermore, most of the diagnostic protocols (e.g. EPPO standards) are based on visual assessment of symptoms or signs on selected susceptible hosts, while alien pathogens often enter new environments through non-hosts, infected asymptomatic native hosts, or associated materials of commerce (e.g. soil, packaging). In fact, as Darwinian evolution predicts, being adapted to and co-evolved with their original native hosts, many of these ‘unknowns to science‘ are unlikely to do noticeable damage on original hosts in their native ecosystems, and are less likely to be detected even when introduced in a new environment with their original native hosts.

In this context it is necessary to develop strategies to describe and study new potential plant pathogens and evaluate the risk they might pose to European flora before their introduction to the old continent. The Sentinel plant concept seems to properly respond to this need. According to this hypothesis, living plant collections cultivated in an exotic environment are exposed to the inoculum pressure of the native pathogens. If a compatible pathogenic interaction occurs, due to the lack of coevolution with the native pathogens, the plants likely will show evident symptoms. The identification of the causal agents of the different symptomatology provides a list of new pathogens potentially harmful to those plants. An International Sentinel Plant Network (ISPN) has been recently developed where living plant collections at botanic gardens and arboreta around the world are networked and serve as early warning systems to help predict and prevent the invasion of new pests (insects, pathogens, or plants) [7]. However, ISPN is dependent on already established exotic plant collections while the efficiency of the strategy relies on the accurate selection of plants to cultivate according to climatic requirements, and the presence of the same genus or family in the exotic area where plantations are established. In order to explore the efficiency of sentinel plantations as an early warning system to prevent invasion of insects and pathogens potentially harmful to EU flora, two experimental plots with European trees were established in China in 2008 [8]. Results regarding colonization of European trees by Chinese insects are presented in a separate paper [9]. This paper reports on the results of the pathogen survey of one of the two plantations by mean of symptoms records, detection of organisms by molecular (mass sequencing) techniques, their taxonomic collocation, and the potential of pathogenicity to EU hosts.

## Materials and Methods

### Sample area

The study was carried out in a sentinel trees plot established near Fuyang (30,003333 N; 119,799722 E; 110m elevation), ca. 40 km southwest of Hangzhou (Zhejiang province, South eastern China), on a small parcel of agricultural land completely surrounded by a mixed conifer- broadleaved forest. The forest was mostly composed of *Pinus massonnianna*, *Cunninghammia lanceolata*, various species of *Fagaceae* and bamboos. The surrounding agricultural land included field crops with an emphasis on rice. The European trees planted in China considered in the study included *Quercus petraea*, *Q*. *Suber* and *Q*. *ilex*. The Fuyang site was planted in May 2008 from one- year old bare-rooted seedlings shipped from Europe to China and kept 3 weeks in quarantine at the port of entry before being planted. Before export to China, the bare-rooted seedlings were individually submitted to thorough phytosanitary inspections by quarantine services to ensure the absence of any insect or pathogen damage, and an additional insecticide and fungicide treatment was applied. The seedlings were planted immediately after being allowed to enter China. The initial height of the seedlings was ca.1-1,5 m- tall at the plantation. Twenty-five seedlings per species were planted in randomized blocks in order to allow statistical analyses. Twice per year from 2008 to 2010, the plot was surveyed for the presence of symptoms and signs of diseases on the base of a visual and, where necessary, microscopic observation of specimens.

At the end of the experiment in autumn 2010, all the seedlings were removed, including roots, and destroyed by burning.

### Characterization of the fungal community of leaves

The detection of the fungal community was performed only on *Q*. *petraea*, *Q*. *suber* and *Q*. *ilex* symptomatic tissues of mature leaves collected in 2010, during the growing season. Samples from each tree were bulked and processed, 1sample/host species.

Genomic DNA was extracted from symptomatic tissue samples in China using the Qiagen DNeasy Plant Mini Kit (Qiagen, Milan, Italy) according to the manufacturer’s protocols. DNA extracts were quantified using the Qubit Quantitation Kit (Life Technologies, Monza, Italy). DNA extraction was carried out in China and sent to DIBAF-University of Tuscia in Italy for library preparation. The non-coding nuclear rDNA ITS region was amplified using the fungal primer pair ITS1F (5’-AxxxCTTGGTCATTTAGAGGAAGTAA- 3’) and ITS2 (5’-BGCTGCGTTCTTCATCGATGC- 3’), where A and B represent the two pyrosequencing adaptors (CCATCTCATCCCTGCGTGTCTCCGACTCAG and CCTATCCCCTGTGTGCCTTGGCAGTCTCAG) and xxx the sample identification barcoding key. The following PCR conditions were used: 94°C for 4 min, 30 cycles of 30 s at 94°C (denaturation), 50°C for 1 min (annealing) and 72°C for 90 s (extension), followed by 10 min at 72°C.

Positive and negative controls were included in the PCR. Amplicons were purified using the Agencourt AMPure XP system (Beckman Coulter Inc., USA) and quantified with the Qubit Quantitation Kit (Invitrogen, USA). The library was sequenced in 1/16 of a standard PicoTitre Plate with the 454 Life Sciences GS-FLX System (Macrogen).

### Bioinformatics and statistical analyses

Raw data were trimmed and analysed using the software CLOTU [10]. Reads with undetermined nucleotide, with any mismatch against tags and primers, and shorter than 150 bp were removed from the data set. Sequence reads from each sample were clustered into similarity-based OTUs (operational taxonomic units) using BLASTClust (single linkage clustering) at 97% similarity and 75% overlap in sequence length between reads in the pairwise alignments [11–14].

One representative sequence from each OTU was submitted to BLASTn [15] for comparison against the GenBank non-redundant (NCBI-nr) database.

OTUs were only assigned to species level if i) the query sequence matched database sequences from fungal isolates (including at least one vouchered specimen) with E-values ≤e−100 and percentage sequence identity ≥97% and ii) at the lowest E-values, when there were no contradictions among different species within the same genus [16]. At lower percentage sequence identity, the query read was identified to genus (identity 95–96%) family (identity 90–94%) order (identity 80–88%) and class level (identity <80%).

The quality of the BLAST-based taxonomic assignment of the OTUs was assessed using two approaches. First, GenBank was queried using the BLASTn algorithm for each sequence, and the top hits recorded. Blast results were inspected manually to remove inconsistencies: sequences were not considered when there was a contradiction in genus assignment in the reference database, since we suspected misidentification of the database sequence. Second, an alignment was generated for each genus using our query sequences, selected top BLASTn hits and the sequence of the first taxonomically named BLASTn hit, if the top hits were unidentified. These alignments were assembled automatically in CLUSTALW [17] with manual editing. Neighbour joining (NJ) analysis, based on K2P distances and tested by 1000 bootstrap replicates (BS), were conducted for each alignment to confirm the identification of query sequence to the genus or species level, using PHYLIP 3.6 [18]. Bayesian analysis was conducted on the same individual dataset used in the NJ analysis. The best nucleotide substitution model was evaluated by MrModeltest v 2.5 [19]. Phylogenetic analysis was performed with Mrbayes v3.1 [20] applying a general time reversible (GTR) substitution model with gamma (G) and proportion of invariable (I) site parameters to accommodate variable rates across sites. Two independent runs of Markov chain Monte Carlo (MCMC) were performed using four chains for 2x10^6^generation and trees were saved each 1000 generations. The initial 250 trees in each run were discarded as “burn-in”. The remaining trees were used to construct majority-rule consensus trees. Bayesian post probabilities (PPs) for each clade were derived from trees remaining after the discarding the burn-in sample. Convergence of runs and parameters was controlled by the diagnostics implemented in MrBayes and in Tracer 1.5 [21].Trees were drawn using TREEVIEW [22].

To assess sampling efficiency, a rarefaction curve was generated by means of the Analytic Rarefaction 1.3 software [23], using 10 specimens as a step parameter for calculation. Statistical analysis of data was performed with Prism version 5.00 software (GraphPad Software, California, USA) and Systat 11 (Systat Software, Inc.).

### Ethics Statement

Specific permissions were not required for these locations and activities and the field studies did not involve endangered or protected species.

## Results

### Characterization of the fungal community of leaves

**Fig 1:**
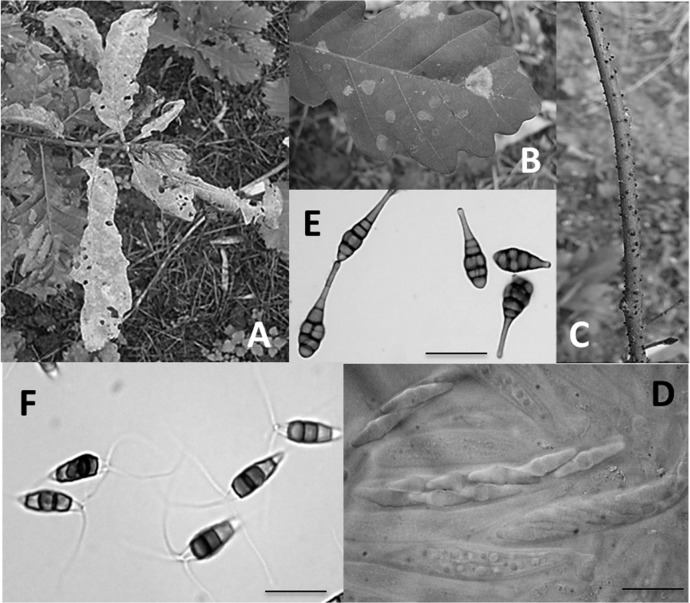
Signs of powdery mildew on leaves of *Q*. *petraea* (A); necrotic spots on leaves of *Q*. *petraea* (B) *Leptosphaeria* sp. perytheciaon branches of *Q*. *petraea* (C); ascopores of *Leptosphaeria* sp. (D); conidia of *Alternaria* sp. observed on specimens of all oak hosts (E); conidia of *Pestalotiopsis* sp. from conidiomata on necrotic spots of *Q*. *petraea* leaves (F). Bars 20 μm.

No symptoms or signs were observed on sentinel oaks in 2008–2009 surveys. However during the 2010 growing season a powdery mildew infection developed with signs consisting of white, talcum powder-like coating on the leaves of *Q*. *petraea*. Sometimes the infected leaves became distorted (Fig. 1A). Leaf spots were also detected on the three *Quercus* species (Fig. 1B). Signs of *Leptosphaeria* cankers were observed on *Q*. *petraea* branches on which perithecia were present (Fig. 1C) containing bitunicate asci with typically3-septate ascospores (Fig. 1D). *Alternaria*-like conidia were also observed on all *Quercus* spp. specimens (Fig. 1E). Asexual fruiting bodies containing *Pestalotiopsis*-like conidia were found in leaf spots of *Q*. *petraea* (Fig. 1F).

**Fig 2:**
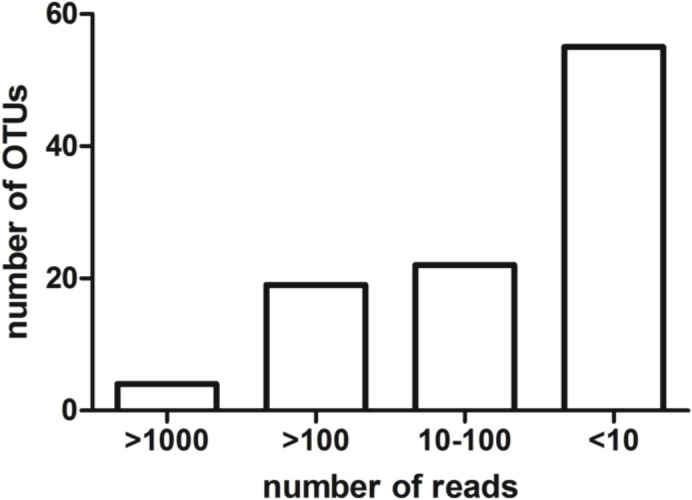
Distribution of number of reads within the 106 identified operational taxonomic units (OTUs), singletons excluded.

After the quality filtering and removal of singletons, considered to be mostly artefacts [14] a total of 14.069 reads were retained for supplementary analyses. Numbers of reads were 746, 2.959, and 10.364 for *Q*. *ilex*, *Q*. *suber* and *Q*. *petraea*, respectively. A total of 106 OTUs distributed among *Q*. *ilex* (30), *Q*. *suber* (59) and *Q*. *Petraea* (93) were considered for further analyses. Four OTUs accounted for the largest fraction of reads (59%) (Fig. 2). Among these, OTU 1 to 3 were ubiquitous on the 3 host species and assigned to *Alternaria* sp., unidentified Basidiomycota and *Epicoccum nigrum* respectively, while OTU4 was specific to *Q*. *petraea* and assigned to *Erysiphe quercicola* (see below).

**Fig 3:**
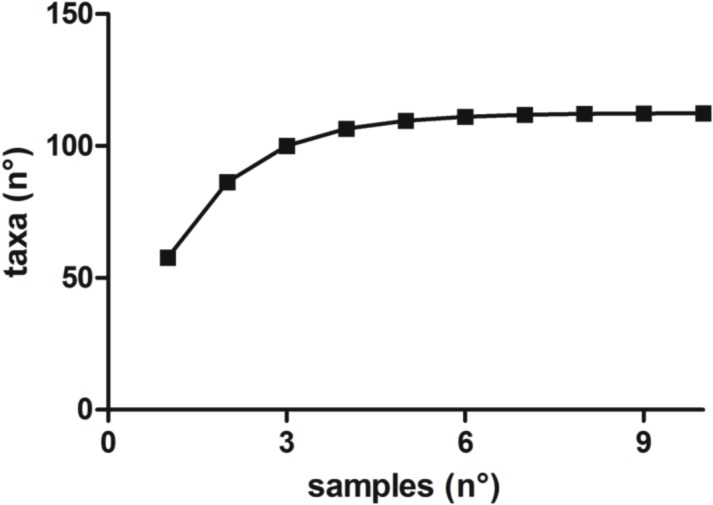
Rarefaction curves describing the observed number of taxa as a function of the number of samples processed.

The plot of OTUs vs the number of samples resulted in a rarefaction curve that approached a plateau (Fig. 3) indicating that the majority of taxa present in the site were covered by the analysis.

A total of 45 out of 106 OTUs (42%) belonged to Basidiomycota, 59 OTUs (56%) to Ascomycota and only 2 OTUs (2%) to Glomeromycota. Dothideomycetes (19%) and Tremellomycetes (26%) were the most frequent classes of Ascomycota and Basidiomycota, respectively.

A total of 9 OTUs have been identified of possible Asiatic origin, based on clustering in NJ trees within groups of sequences of taxa mostly reported from Asia.

Among these species only 2 belong to Basidiomycota:

OTU91, based on the phylogenetic analysis (BS = 88 and PPs = 95) was ascribed to Fungal sp. NW-AN003, belonging to the genus *Cryptococcus* and previously recorded as endophyte of larch in China (NCBI accession number JN809912);

OTU 48 was assigned to *Cryptococcus* sp.; the best blast hits of this read consisted in 8 isolates of *Cryptococcus* sp., from plants leaves in China (NCBI accessions numbers: HQ890368, HQ890367, HQ890366, HQ890365, HQ890364, HQ890363, HQ890362, HQ890369) (BS = 99 and PPs = 99);

Six OTUs were assigned to Ascomycota

**Fig 4:**
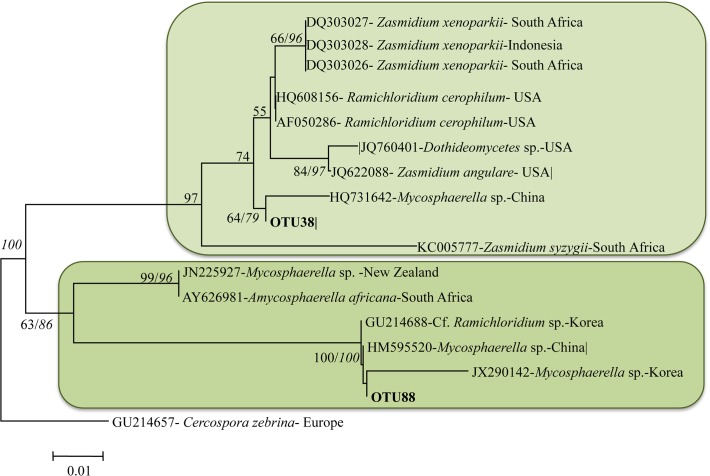
Phylogenetic relationship between OTU88, OTU38 and top BLAST hits species (with GenBank accession numbers and geographic distribution). The tree was constructed using the neighbor-joining cluster algorithm. Bootstrap support (%) and posterior probabilities are provided on the branches (BS/PP). Bootstrap values below 50 are not shown. Scale bar, 0.002 substitutions/site.

OTU88 and OTU38 were ascribed to the genus *Mycosphaerella* (Fig. 4):

OTU88, was detected on *Q*. *petraea* and *Q*. *suber*. Itforms a monophyletic clade with Cf. *Ramichloridium* (NCBI accession number GU214688), *Mycosphaerella* sp. M16 (NCBI accession number HM595520) and *Mycosphaerella* sp.C 2–2 (NCBI accession number JX290142) (BS = 100 and PPs = 100), isolated from *Phellodendron amurense* in South Korea *Abies beshanzuensis* in China and *Toona sinensis* in Korea, respectively [24], [25]. OTU38 was detected on *Q*. *ilex*, representing 3.2% of the total reads, and, at lesser extent, on *Q*. *petraea* and *Q*. *suber*. It has been identified as *Mycosphaerella* sp. Ponipodef 06 (NCBI accession number HQ731642) isolated from *Populus nigra* x *P*. *deltoides* in China (BS = 88 and PPs = 60).

OTU25 was detected on *Q*. *petraea* and *Q*. *suber* (and other hosts, data not shown) and assigned to *Strelitziana albiziae* (NCBI accession number HQ599584) isolated from leaves of *Albizia julibrissin* infected with *Camptomeris albiziae* in South Korea [26].

**Fig 5:**
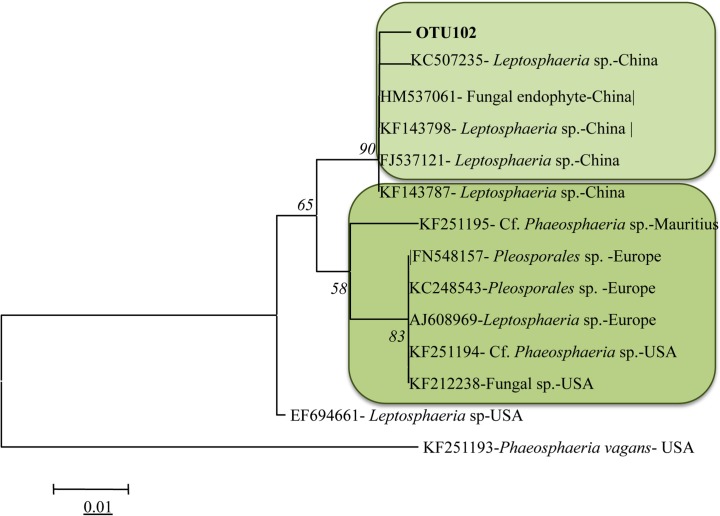
Phylogenetic relationship between OTU102 and top BLAST hits species (with GenBank accession numbers and geographic distribution). The tree was constructed using the neighbor-joining cluster algorithm. Bootstrap support (%) and posterior probabilities are given on the branches (BS/PP). Bootstrap values below 50 are not shown. Scale bar, 0.002 substitutions/site.

OTU102 was detected on *Q*. *petraea* and identified as *Leptosphaeria* sp. It formed a well-supported clade (BS = 93 and PPs = 97) including only isolates from China (NCBI accession numbers:HM537061,KF143798,FI537121,KF143787) (Fig. 5).

**Fig 6:**
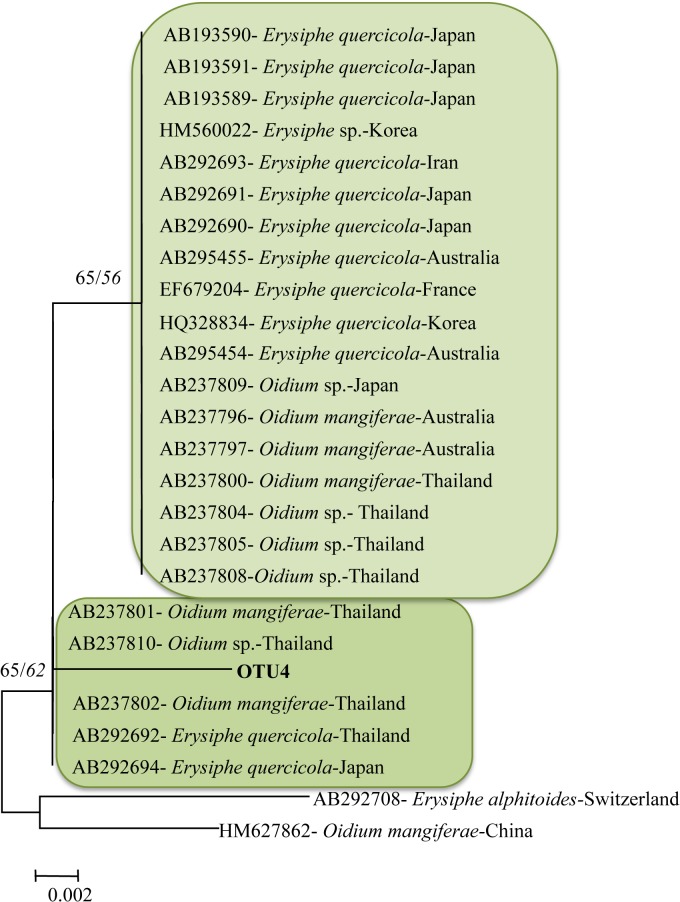
Phylogenetic relationship between OTU4 and top BLAST hits species (with GenBank accession numbers and geographic distribution). The tree was constructed using the neighbor-joining cluster algorithm. Bootstrap support (%) and posterior probabilities are given on the branches (BS/PP). Bootstrap values below 50 are not shown. Scale bar, 0.002 substitutions/site.

The blast and the phylogenic analysis assigned OTU4 to *Erysiphe quercicola*. No additional OTUs related to Erysiphales have been identified. From the phylogenetic analysis it is clear that OTU4 is closely related to two isolates from Asia (Thailand and Japan). The division between OTU4 in one clade and the isolate from France on another clade is well supported (BB = 66 and PPs = 81) (Fig. 6).

OTU77 showed 97% (E-value = 3e-95) pairwise similarity to *Lalaria* sp. JS-42 (NCBI accession number JF706660) isolated from flowers in Korea (BB = 100 and PPs = 97).

In addition, OTU16 was assigned to *Pestalotiopsis* sp. (97% similarity; E-value = 3e-100). It accounts for about 2.5% of the total reads on *Q*. *petraea*. Although the Asiatic origin of this OTU cannot be demonstrated, it probably refers to a new species.

**Fig 7:**
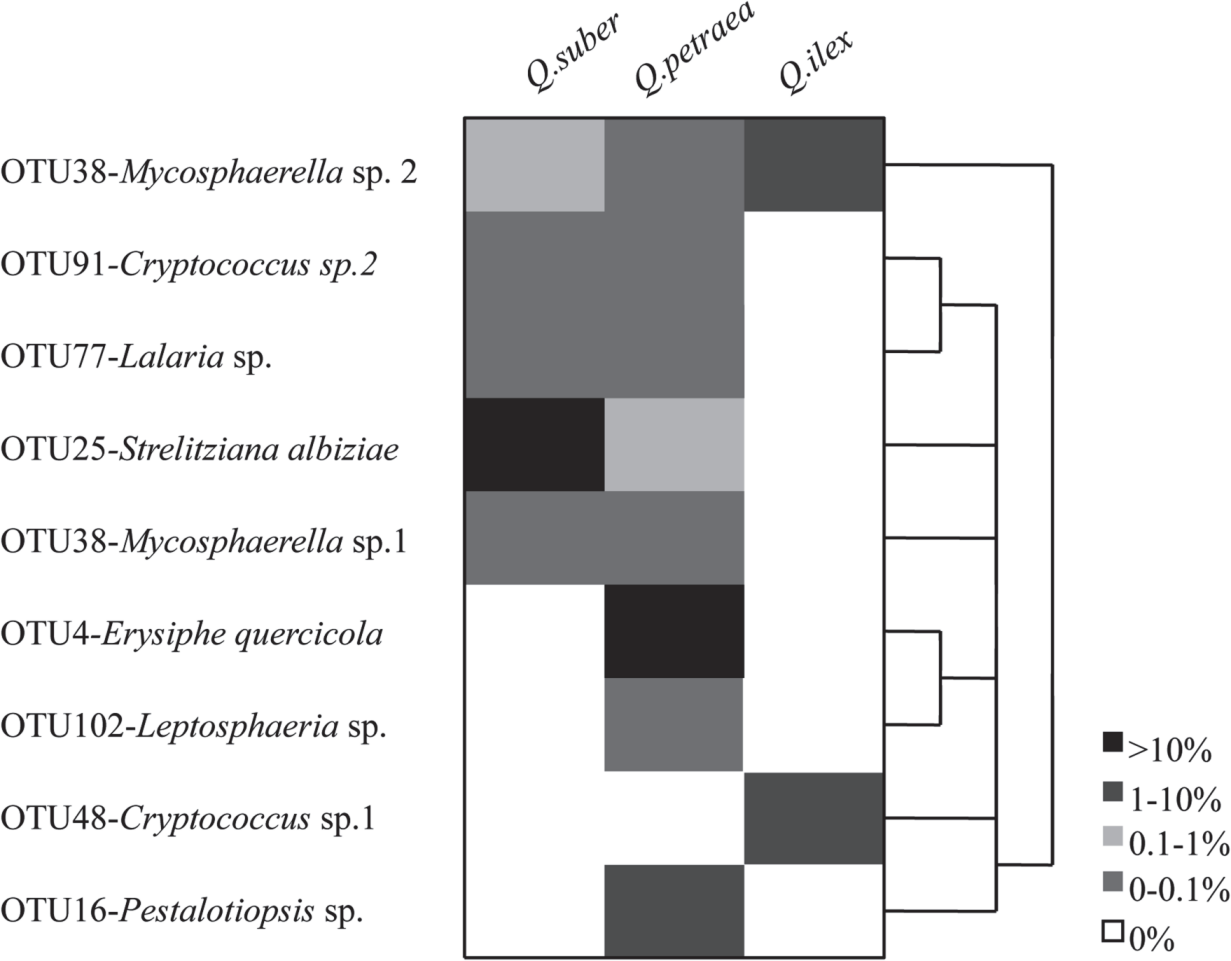
Hierarchically clustered heat map of the taxa distribution with Asian origin. The relationship among samples was determined by Bray-Curtis distance. The heat map plot depicts the relative percentage of each fungal taxa (variables clustering on the vertical-axis) within each tree host (horizon-axis clustering). The relative abundance for fungal taxa are indicated by color intensity with the legend indicated at the bottom of the figure.

A graphical representation of OTUs abundance and distribution among hosts is showed in Fig. 7.

In Table 1, the information about identification of taxa and association with signs and symptoms are summarized.

**Table 1:** Summary table on identification and association to signs and symptoms of the OTUs of possible Asiatic origin.

N°	OTUs	Taxa	Identification		Association with signs and symptoms
			Molecular	Morphological	
1	4	*Erysiphe quercicola*	YES	YES	YES
2	16	*Pestalotiopsis* sp.	YES	YES	YES
3	25	*Strelitziana albiziae*	YES	NO	NO
4	38	*Mycosphaerella* sp. 1	YES	NO	NO
5	48	*Cryptococcus* sp.*1*	YES	NO	NO
6	77	*Lalaria* sp.	YES	NO	NO
7	88	*Mycosphaerella* sp.	YES	NO	NO
8	91	*Cryptococcus* sp.2	YES	NO	NO
9	102	*Leptosphaeria* sp.	YES	YES	YES

## Discussion

Our study reports for the first time the colonization of a selected collection of EU trees by native fungi while growing in China, and the potential of sentinel plantations to detect taxa pathogenic to EU trees that might pose a biosecurity risk for Europe. The sentinel plantation of Fuyang represents an improvement of the ISPN hypothesis based on the use of existing botanical gardens worldwide. Infact, in this study, the EU trees were selected according to ecological criteria. They were planted in China in sites comparable in climate and plant community composition to those of their area of origin in Europe. Presence of native species belonging to the same family or genus (e.g. the *Fagaceae* family in the Fuyang plot) and having similar ecological requirements enhances the probability of exposure of sentinel trees to natural inoculum pressure. The placing of the European trees in these specific sites was thought to favours “host jump” by native pathogens facilitating the evaluation of their aggressiveness.

Four out of the nine taxa of Asiatic origin, are related to plant pathogenic genera and were associated with symptoms and signs typical of infection on the sentinel trees.

The OTU 38 and 88 refer to the *Mycosphaerella* complex (Fig. 4) and specifically to fungi isolated from woody hosts other than *Fagaceae* in Asia. Specifically OTU88 refers to taxa isolated from *Abies beshanzuensis* and *Toona sinensis* host species present in the Fuyang forest areas, and plantations (*Phellodendron amurense*). *Phellodendron amurense* is native of Northeastern provinces of China but it is widely cultivated in the Zhejiang provinces. Furthermore, this host is reported to be impacted by at least 3 anamorphic *Mycosphaerellaceae* in China [27] although no sequence data are available for comparison. OTU38 refers to taxa isolated from leaves of *Populus x Euroamericana* also cultivated in the Zhejiang province. These 2 OTUs were identified from leaf spots of sentinel *Quercus petraea*, *Q*. *suber* and *Q*. *Ilex* plants. Additional OTUs (37 and 44) referring to the *Mycosphaerella* anamorphs *Cercospora* and *Pseudocercosporella* were identified and also associated to leaf spots of sentinel oak trees. However their Asiatic origin could not be proved. *Mycosphaerella* spp. are among the most common and destructive plant pathogens known, causing considerable economic losses on a wide variety of host plants worldwide, including economically important horticultural and ornamental crops [28], [29]. The term *Mycosphaerella* is linked to more than 30 anamorphic form genera and account for several thousand species (ca 10.000) most of which are plant pathogens [24]. Interestingly, Crous [24] in his review on *Mycosphaerella* complex, argues that most of the investigated host plants are associated to several undescribed taxa in the *Mycosphaerella* complex. Many of these species can colonise the same lesion, have the ability to appear in non-host tissue, and to actively undergo host shifts while infecting plant tissue. In such context *Mycosphaerella* spp. are perfect candidates to become invasive in new environments on new hosts. In addition *Quercus* is already reported as a common host of several *Mycosphaerella* species; Gilman and Wadley [30] listed at least 8 species pathogenic on oaks in the USA.

OTU 102 refers most probably to a Chinese *Leptosphaeria* sp. as supported by phylogenetic analysis (Fig. 5). The closest sequences were assigned to an undescribed *Leptosphaeria* sp. isolated from plant specimens in China [31] The genus *Leptosphaeria* includes a large number of species mainly considered saprobic or necrotrophic on stems and leaves. The genus also comprises highly pathogenic species such as *L*. *maculans* and *L*. *biglobosa*, the causal agents of ‘blackleg’ of *Brassicae* crops [32], and *L*. *coniothyrium* causal agent of stem canker of raspberry and roses [33]. The association of typical symptoms and signs with molecular detection by mass sequencing suggests the interaction of *Leptosphaeria* sp. with *Q*. *petraea*, although the pathogenic behaviour must be demonstrated.

The finding of the biotrophic pathogen *E*. *quercicola* on *Q*. *petraea* is of interest because this species has been described from *Q*. *phillyraeoides* by Takamatsu et al. [34]. It was described as a new species genetically and morphologically distinct from *E*. *alphitoides* and having an Asiatic and Australian distribution. Most recently *E*. *quercicola* has been detected in France and associated with flag-shoot symptoms of *Q*. *robur* and *Q*. *petraea* [35]. Mougou et al. [36] suggested that *E*. *quercicola*, similar to other oak powdery mildews in Europe, was introduced on European oaks through host shift from traded exotic host species such as *Q*. *phillyraeoides* (present in many nursery catalogues in France). Furthermore Kirshner and Liu [37] reported anamorphic *E*. *quercicola* on 2 tropical hosts commonly traded to Europe as ornamentals, *Cinnamomum camphora* and *Murraya paniculata* highlighting the risk of host shift from taxonomic distant species. These native hosts, including *Q*. *phillyraeoides*, are common in the Fuyang area supporting the presence of source of natural inoculum of *E*. *quercicola*. In the sentinel plantation studied, signs and symptoms of powdery mildew were observed in 2010 (third year from establishment of the plantation) on *Q*. *petraea* but not on *Q*. *suber* and *Q*. *ilex*. The molecular detection confirmed the presence of *E*. *quercicola* limited to *Q*. *petraea* (1214 reads). Moreover, the phylogenetic analysis confirms that OTU4 is clearly distinguished from the sequence detected in France (Fig. 5). The recovery of *E*. *quercicola* on *Q*. *petraea* in the Fuyang sentinel plantation represents an “a posteriori” demonstration of the efficacy of such an approach to detect plant pathogens cable of invading and causing negative impacts to European forests.

The detection of *Pestalotiopsis* sp. is particularly interesting. While no specific signs of *Mycosphaerella* (reproductive structures, spores) have been reported from specimen observations, conidiomata of *Pestalotiopsis* sp. and associated conidia were observed on *Q*. *petraea* leaf spots. Species of *Pestalotiopsis* commonly cause diseases on a variety of plants. They are also frequently isolated as endophytes or occur as saprobes [38]. In such context the presence of conidiomata of *Pestalotiopsis* on necrotic tissues does not necessarily demonstrate the pathogenic behaviour of this taxa to *Q*. *petraea*. However, the low value of identity of OTU16 (97%) with the sequences of *Pestalotiopsis* spp. in the NCBI database and its considerable presence on *Q*. *petraea* (2.5% of the total reads) support the hypothesis of a new species not previously recorded.

Although the potential of sentinel plantations to intercept local plant pathogens seems high, several bottlenecks need to be resolved before this approach can be routinely utilized. The Fuyang plantation was established with 1–2 year old seedlings shipped directly from Europe. This sentinel plantation was primarily designed to facilitate the detection of damaging pests (insects). In this context, the choice of propagation material for the plantation dealt only with the need of having medium sized trees in place for insect pests attack. However, the type of propagation material gains importance when fungal (or other plant pathogens) detection is considered. The use of seedlings imported from Europe to establish the sentinel plantation does not eliminate the risk of their colonization by fungal taxa at the area of production. To circumvent this, the use of certified seeds to establish the plantation is recommended in order to minimize the risk of endophytic infection by European taxa. In any case, a characterization of possible microbial community residents in the propagation material is mandatory before the plantation is established to provide a baseline for colonization by native organisms in the sentinel plantation. In this study the detection of putative Chinese fungi infecting the European sentinel trees was performed by symptom/sign assessment and molecular methods. Both approaches are appropriate and necessary, but certainly not sufficient to prove pathogenicity, especially for taxa behaving both as saprophyte and pathogens. While the assessment of specific signs and symptoms coupled with the molecular detection are exhaustive for biotrophic pathogens, like *E*. *quercicola*, the presence of different *Mycosphaerella* spp. associated with leaf spots could be explained by both a pathogenic and saprophytic interaction. Biological characterization of the organisms associated with the disease and the reproduction of symptoms through inoculation trials on healthy hosts is required to prove pathogenicity and should always follow the molecular characterization. Obviously, sentinel plant material suspected to be infected by native plant pathogens cannot be shipped abroad and should be processed in specialised laboratories “in loco”. In this study symptomatic material was processed ‘in loco’ for DNA extraction and purification; unfortunately biological detection and isolation of candidate pathogenic fungi failed and could not be completed due to bottlenecks in collaborative agreements. For the complete success of a sentinel tree strategy, the quality of reciprocal agreement between the collaborating countries including shared protocols for plantation establishment and samples processing is of importance.

In conclusion, the sentinel plantations represent an appropriate answer to the growing need of preventive identification and characterization of plant pathogens at risk of introduction and invasion in Europe (and elsewhere). However the preliminary experience carried out in the Fuyang plantation highlighted the logistic and technical difficulties in utilizing sentinel trees for pathogens detection. Efforts should be made to solve regulatory and logistic bottlenecks associated with biosecurity issues and the possibility of full characterization of biological samples, in order to make possible the routinary and reciprocal utilization of this approach between countries.
